# Assembling large genomes: analysis of the stick insect (*Clitarchus hookeri*) genome reveals a high repeat content and sex-biased genes associated with reproduction

**DOI:** 10.1186/s12864-017-4245-x

**Published:** 2017-11-16

**Authors:** Chen Wu, Victoria G. Twort, Ross N. Crowhurst, Richard D. Newcomb, Thomas R. Buckley

**Affiliations:** 1 School of Biological Sciences, The University of Auckland, Auckland, New Zealand; 2 Landcare Research, Auckland, New Zealand; 3 New Zealand Institute for Plant & Food Research Ltd, Auckland, New Zealand; 4 Department of Biology, Lund University, Lund, Sweden

**Keywords:** Phasmatodea, Genome assembly, RNA-Seq, Reproduction, Parthenogenesis, *Clitarchus hookeri*

## Abstract

**Background:**

Stick insects (Phasmatodea) have a high incidence of parthenogenesis and other alternative reproductive strategies, yet the genetic basis of reproduction is poorly understood. Phasmatodea includes nearly 3000 species, yet only the genome of *Timema cristinae* has been published to date. *Clitarchus hookeri* is a geographical parthenogenetic stick insect distributed across New Zealand. Sexual reproduction dominates in northern habitats but is replaced by parthenogenesis in the south. Here, we present a de novo genome assembly of a female *C. hookeri* and use it to detect candidate genes associated with gamete production and development in females and males. We also explore the factors underlying large genome size in stick insects.

**Results:**

The *C. hookeri* genome assembly was 4.2 Gb, similar to the flow cytometry estimate, making it the second largest insect genome sequenced and assembled to date. Like the large genome of *Locusta migratoria*, the genome of *C. hookeri* is also highly repetitive and the predicted gene models are much longer than those from most other sequenced insect genomes, largely due to longer introns. Miniature inverted repeat transposable elements (MITEs), absent in the much smaller *T. cristinae* genome, is the most abundant repeat type in the *C. hookeri* genome assembly. Mapping RNA-Seq reads from female and male gonadal transcriptomes onto the genome assembly resulted in the identification of 39,940 gene loci, 15.8% and 37.6% of which showed female-biased and male-biased expression, respectively. The genes that were over-expressed in females were mostly associated with molecular transportation, developmental process, oocyte growth and reproductive process; whereas, the male-biased genes were enriched in rhythmic process, molecular transducer activity and synapse. Several genes involved in the juvenile hormone synthesis pathway were also identified.

**Conclusions:**

The evolution of large insect genomes such as *L. migratoria* and *C. hookeri* genomes is most likely due to the accumulation of repetitive regions and intron elongation. MITEs contributed significantly to the growth of *C. hookeri* genome size yet are surprisingly absent from the *T. cristinae* genome. Sex-biased genes identified from gonadal tissues, including genes involved in juvenile hormone synthesis, provide interesting candidates for the further study of flexible reproduction in stick insects.

**Electronic supplementary material:**

The online version of this article (10.1186/s12864-017-4245-x) contains supplementary material, which is available to authorized users.

## Background

The insect order Phasmatodea, commonly known as stick insects or walking sticks, contains approximately 3000 species distributed worldwide [1]. At least 10% of stick insect species can reproduce parthenogenetically and for this reason have attracted much attention [1]. *Clitarchus hookeri* is one of the most common New Zealand stick insect species and is distributed across a wide range of habitats on both the North and South Islands with a lower population density at higher latitudes and altitudes [2]. Its reproductive biology is interesting as it displays extreme sexual dimorphism and geographical parthenogenesis [2, 3]. On the upper half of the North Island sexual reproduction dominates, which produces offspring with relatively equal numbers of both sexes, whereas, obligate parthenogenesis is widespread on the lower North Island and South Island, forming all-female populations [2, 3]. In addition, *C. hookeri* is also thought to have hybridized with the obligate parthenogenetic genus *Acanthoxyla* [4–7]. All these features make *C. hookeri* an ideal species for the study of geographical parthenogenesis, hybridisation, and mating behaviour [2–5, 7–13]. However, at the molecular level, little is known about these processes, impeding further understanding of their reproductive biology. Currently, except for some genes encoding male accessory gland proteins [12] from *C. hookeri*, the genes involved in other female and male reproductive traits, such as oogenesis, spermatogenesis, egg and sperm maturation are largely unknown. It is critical that these genes are characterised to advance our understanding of the evolution of parthenogenesis and other alternative reproductive processes within the Phasmatodea.

RNA-Seq based transcriptome profiling has been successfully employed to identify genes expressed in the germline tissues from both model and non-model insect species, such as *Aedes aegypti* (mosquito) [14], *Nasonia vitripennis* (wasp) [15] and *Periplaneta americana* (cockroach) [16]. By sampling gonadal tissues from males and females, a digital gene expression profile can be obtained from the RNA-Seq data for the identification of the genes predominantly expressed in one sex (sex-biased genes). These genes frequently encode proteins essential to the sex-linked characteristics that are critical to the study of reproduction and its evolution [17]. Coupling genome and transcriptome sequencing allows the use of a reference-guided approach to studying patterns of gene expression. This is thought to be more accurate than the de novo method, because it can reduce transcript redundancy, reveal strand orientation to tease apart overlapping transcripts and enhance the representation of lowly expressed genes [18].

Another interesting feature of the stick insects is their large genome size. Currently, only the genome of *Timema cristinae*, sister lineage to all other stick insects (Euphasmatodea), has been sequenced and assembled [19]. However, compared with the 1.5 Gb genome of *T. cristinae*, even larger genomes occur in Euphasmatodea. Multiple species from the European stick insect genus *Bacillus* are ~2 Gb [20], while the South American species *Anisomorpha buprestoides*, Lord Howe Island species *Dryococelus australis* and the Australian species *Extatosoma tiaratum* have even larger genomes, up to ~3 [21], ~4 [22] and ~8 Gb (reported from the Animal genome size database: http://www.genomesize.com/), respectively. Without genome sequences from euphasmids, we are unable to determine the main causes for the formation of these large genomes. High-throughput sequencing has been used to sequence the whole genomes of some model and non-model species, especially those from the more derived Holometabola with small or moderate genome sizes [23–27]. Recently, the *Locusta migratoria* (Orthoptera) genome sequence comprising 6.5 Gb has become available. The analysis of this genome revealed a large numbers of repetitive elements (~58.9% of the genome assembly) and gene copy expansion within some gene families (e.g. detoxification) [28]. This resource has enhanced our understanding of the causes and consequences of large insect genomes, but whether similar patterns also occur in other insect lineages with large genomes is unknown.

Here we have sequenced, assembled and analysed the genome of *C. hookeri*, and through comparison with other insects, provide insights into the evolution of stick insect genomes. We particularly focused on repetitive elements and predicted gene models, both potential factors underlying large euphasmid genome sizes. We also performed an analysis of the RNA-Seq data produced from sequencing the female reproductive tract and the male testis to identify genes that are essential to gamete production and maturation in *C. hookeri*. The candidate genes predominantly expressed in the gonad of one sex and the genes involved in the juvenile hormone pathway provide candidates for the further study of stick insect reproductive flexibility.

## Methods

### Sample collection and preparation

All samples used for sequencing in this study were collected from Totara Park, Auckland, New Zealand (37°0.111 S, 174° 55.039 E). A female *C. hookeri* (CLI739) was collected in 2013 and leg tissues were used for the estimation of genome size. Three female insects (CLI525, 600 and 654) were collected in 2012 for genome sequencing and leg tissues were used. Three males (CLI755, CLI757 and CLI760) and three females (CLI765, CLI767 and CLI768) were collected in 2014 for gonadal transcriptome sequencing. Females were nymphs when collected and separately reared from males until they laid the first egg (reached maturation). Live insects were snap frozen in liquid nitrogen on capture and stored at −80 °C after collection. Female reproductive tract (approximately 18 to 20 ovarioles, early developing eggs and oviducts), and male testicle pairs were dissected in ethanol (100%) for RNA extraction.

### Genome size estimation

The genome size of *C. hookeri* was estimated using flow cytometry following the Otto two-step method with the substitution of propidium iodine for DAPI [29]. A female *L. migratoria* was used as an internal standard. Approximately 15 mm^2^ of leg tissue with a standard was co-chopped in a few drops of ice cold Otto buffer 1 with a stainless steel razor blade and then incubated for approximately 2 min. The sample was then filtered through a 20 μm Celltrics filter (PARTEC GmbH) before adding 2.5 ml of Otto buffer 2 with 1 mg/ml propidium iodine. The sample was run on a PARTEC CyFlow Space with a 488 nm laser as the excitation source. The 2C content of the *L. migratoria* standard was determined to be 18.31 pg, using *Pisum sativum* Citrad (9.09 pg 2C content; 4445 Mbp) [30] internal standard, and the gain adjusted as required. The total amount of DNA in the sample was determined as the ratio of the average channel number of the sample 2 N to the average channel number of the standard 2 N times the 1C amount of DNA in the standard.

### DNA extraction, library preparation and sequencing

Genomic DNA was extracted from *C. hookeri* leg tissue using the DNeasy Plant Mini kit (Qiagen) with the following modifications: frozen leg tissue was chopped and incubated for 1 h with digestion buffer before DNA elution columns were incubated with digestion buffer for 1 h, and after the addition of elution buffer columns were incubated at room temperature for 30 min prior to centrifugation. DNA was quantified by a Nanodrop 2000 spectrophotometer (Thermo Fisher Scientific) and quality checked by running a sample on 0.5% *w*/*v* agarose gel stained with 1X GelRed (Huntingtree Ltd.). The resulting nuclear genomic DNA was sent to New Zealand Genomics Limited (NZGL: http://www.nzgenomics.co.nz/) Otago, Dunedin, for library construction and sequencing. Seven paired-end (PE) sequencing libraries with average insert sizes of 200 bp (2 libraries), 350 bp, 500 bp and 720 bp (3 libraries) (Table 1) were prepared using the TruSeq® DNA LT Sample Prep Kit v2 (catalogue ID: FC-121-2001) and sequenced with 11 lanes on an Illumina HiSeq2000™. The three mate-pair (MP) libraries with insert sizes of 5 k bp and 8 k bp (2 libraries) (Table 1) were constructed using the Nextera® Mate Pair Sample Prep Kit (catalogue ID: FC-132-1001) and sequenced with eight lanes on the same platform. The number of libraries per insert size and the number of lanes sequenced per library are described in Table 1. All reads were 101 bases in length. For RNA-Seq, total RNA extraction and library preparation were performed as described in [12]. The extractions were barcoded and then pooled together for sequencing on the HiSeq2000 platform for one lane to generate 100 bp PE reads at NZGL.

**Table 1:** Illumina sequencing output for *Clitarchus hookeri* whole genome assembly

Insert size (bp)	Sample × library × lane	Sequencing output (Gb)	Estimated Genome coverage (×)
200	CLI525 × 1 × 5, CLI600 × 1 × 1	261.2	59.4
350	CLI525 × 1 × 2	88.5	20.1
500	CLI600 × 1 × 1	39.7	9.0
720	CLI525 × 1 × 1, CLI600 × 2 × 2	72.3	16.4
5000	CLI654 × 1 × 4	126.4	28.7
8000	CLI525 × 1 × 2, CLI654 × 1 × 2	145.2	33.0
	Total	733.3	166.6

Genome coverage estimates were obtained using the flow cytometry genome size

### De novo genome assembly and quality assessment

The raw PE reads were preprocessed to remove duplicate sequence pairs, possible contaminants, and low-quality bases as follows: 1) reads containing ambiguities (Ns) and duplicates were filtered using PRINSEQ (v0.20.3) [31] and FastUniq (v1.1) [32], respectively; 2) reads with adapters and low quality ends (Phred < 30) were trimmed using Cutadapt (v1.3) [33]; 3) a read pair with overlapping ends more than 10 bp was merged into a single read using “abyss-mergepairs” from ABySS (v1.5.1) [34]; 4) reads shorter than 50 bp and orphan reads (single pair) were discarded; 5) remaining reads (83.3%) were error corrected using “ErrorCorrectReads.pl” from ALLPATH-LG (v46436) [35]. The raw MP reads were preprocessed following 1), 2), 4), and then trimmed to retain the first 36 bases of the 5 prime end in order to minimise inclusion of Nextera® adapters resulting from library preparation.

*De bruijn* graph and initial contigs were constructed and assembled using “pregraph” and “contig” commands from SOAPdenovo2 (vR223) [36] with Kmer 75 on the PE data derived from a single insect (CLI525; Table 1). The resulting contigs were used to construct scaffolds using “map” and “scaff” commands from SOAPdenovo2 based on their relationships implied by mapping all PE reads to the contigs from the shortest to the largest insect size libraries. Sequence gaps were then filled by GapCloser (v1.12-r6) [36] with PE reads from CLI525, and the resulting sequences were re-scaffolded again by non-CLI525 PE reads using SSPACE-basic (v2.0) [37] with default options (−z 0, −k 5, −a 0.7 and −n 15) and CLI525 PE reads with additional option “−X 1” for extending scaffolds. SSPACE-basic was also used to post-scaffold the resulting scaffolds using MP reads from the shortest to the largest insert size libraries with default options, followed by a step of gap filling using CLI525 reads, as described above, to produce the final genome assembly.

Approximately 1.0 billion genomic PE reads and 54.7 million RNA-Seq reads [12] were mapped to the genome assembly using Bowtie2 (v2.2.0) [38] with paired-end mode (−q −1 R1.fastq −2 R2.fastq) to produce a proportion of the coverage of the assembly from the short reads. The assembly was then quality evaluated with Core Eukaryotic Genes Mapping Approach (CEGMA; v2.4) [39] with large genome size mode (−mam) and Benchmarking Universal Single-Copy Orthologs (BUSCO; v2.0.1) [39] with searching database “arthropoda_odb9” to detect the presence of a core protein set of 248 highly conserved eukaryotic genes and 1066 highly conserved arthropoda genes. Scaffolds were also searched against GenBank *nt* database (Release 212) to estimate the percentage of archea, bacteria and virus sequences.

### Repeat identification

RepeatModeler (v1.0.8) [40] with default options was used to predict and classify repetitive elements. It employs two de novo repeat finding programs, RECON and RepeatScout, to identify repeat element boundaries, followed by an assignment to the repeat classes based on the sequence feature.The resulting repeat models were searched against GenBank non-redundant (*nr*) protein database (evalue <10^−5^) using Blastx (v2.2.28) [41] to exclude potential protein-coding genes. An additional repeat classification was conducted using PASTEClassifier (v1.0) [42] to assign miniature inverted repeat transposable elements (MITEs). The abundances of all predicted repeats were estimated in the genome assembly with RepeatMasker (v4.0.5) [43] (−no_is, −gff and −lib RepeatModels.gff).

### Gene model annotation

Structural gene annotation was performed using MAKER2 (v2.31.3) [44] on scaffolds longer than 2000 bp. Before annotation, meta parameters of *C. hookeri* protein-coding genes, including those determining intron and exon length distributions, splice site patterns, and translation start codon patterns were generated using AUGUSTUS (v3.0.2) [45] (optimize_augustus.pl). Spliced alignments of protein sequences as inputs for AUGUSTUS were generated by aligning *C. hookeri* protein sequences including CEGMA predicted proteins and those that were identified from the head and prothorax transcriptome [9] to the assembly using Scipio (v1.4.1) [46] with default options. The workflow of MAKER2 involves: 1) producing *ab initio* gene predictions using trained *C. hookeri* meta parameters, 2) aligning de novo transcripts collected from [9], [12] and Arthropoda conserved protein sequences (OrthoDB: v7) [47] to the assembly followed by the identification of intron-exon boundaries and splice forms as evidence, 3) producing evidence-informed gene predictions, computing quality scores and selecting the gene models best supported by the evidence. The resulted *C. hookeri* gene models were searched for the presence of core proteins (BUSCO) and homology matches against *nr* (evalue <10^−5^) for gene annotation. These gene models were also identified with *T. cristinae* orthologues from a reciprocal blast method using a custom python script. The *T. cristinae* gene models (v0.2) were downloaded from: http://nosil-lab.group.shef.ac.uk/?page_id=25.

### Transcript construction and annotation

Raw reads were trimmed 5′ end (8 bases), adapter sequences, low quality 3′ ends and filtered reads containing ambiguous bases (Ns) using PRINSEQ (v0.20.3) [31] and CUTADAPT (v1.3) [33]. The program STAR (v2.5) [48] with options “−−outFilterType BySJout −−outFilterIntronMotifs RemoveNoncanonical −−outSAMstrandField intronMotif −−outSAMtype BAM SortedByCoordinate −−outReadsUnmapped Fastx” was used to align reads to the genome assembly (scaffolds longer than 10 kbp). We did not include the putative gene models generated from the in silico gene annotation above as the reference gene set for annotating the gonad-expressed genes; instead, the regions mapped by all the RNA-Seq reads were counted as the gene loci. Cufflinks (v2.2.1) [49] with default options was used to generate these gene loci on the genome assembly according to the alignments, followed by producing merged gene loci using an embedded command “cuffmerge”. The gene loci present with intron-exon boundaries were stranded on the genomic scaffolds. The transcript set was constructed by extracting sequences from annotated gene loci using “gffread”. For multiple isoforms detected from a single gene locus, the first (longest) isoform present was chosen as the representative transcript that was subjected to a search against the SwissProt (release-2015_12) [50], UniProt (release-2015_12) [51] and Flybase (*Drosophila melanogaster*: dmel_r6.08) [52] protein databases using BLASTx (v2.2.28, E-value cut-off: 10^−5^, keeping the top hit). These isoforms were also searched against GenBank *nt* (release14) using Blastn (evalue < 10^−10^) to screen for contamination from bacteria, fungi and virus.

### Read quantification and differential expression analysis

The RNA-Seq reads were aligned to the annotated gene loci followed by quantification. The number of read pairs aligned to the stranded loci was calculated using htseq-count from HTSeq (v0.6.1) [53] with default options and the unstranded gene loci with an additional option “−s no”. Differential expression comparison was performed in R (v3.1.1) [54] using the DESeq2 Bioconductor package [55]. This program takes read counts to estimate sample size factors, followed by estimating dispersions with expected mean values from the maximum likelihood estimate of log2 fold changes, and then fits a negative binomial distribution [55]. The principle component analysis from DESeq2 and an R package pheatmap were used to visualise global similarities and differences. Transcripts with an adjusted *p* value less than 0.05 and a minimum fold change (FC) of 2 were reported as differently expressed.

### Gene ontology and pathways analysis

The predicted gene loci showing significantly sex-biased expression were used to detect enriched gene ontology (GO) terms and Kyoto Encyclopedia of Genes and Genomes (KEGG) pathways. The matched *D. melanogaster* FlyBase gene IDs were imported into the Database for Annotation, Visualization, and Integrated Discovery (DAVID, v6.8) [56, 57] for functional annotation and enrichment tests. The default FlyBase database was used as the background. The level one GOs (GOTERM_1) and the GOs directly mapped (GOTERM_DIRECT) from the source database (without parental terms) were generated with *p* values below 0.05, and the significantly enriched ones were defined according to the *p* values adjusted by the Benjamini and Hochberg (BH) procedure (<0.05). They were visualised using the R package GOplot [58]. The enriched KEGG pathways (KEGG_PATHWAY) were generated with a *p* value less than 0.1.

## Results

### Genome sequence and analysis

The genome size of *C. hookeri* was estimated at approximately 4.4 Gb using flow cytometry. Illumina reads derived from ten libraries with various insert sizes shown in Table 1 were subject to de novo assembly. Sequencing of multiple libraries derived from DNA of a single female (CLI525) yielded 400.4 Gb of PE reads for contig construction with an estimated coverage of 90.9 × the estimated genome size and the initial contigs constructed from these data had a N50 of 3715 bp. The final assembly had a total size of 4.2Gb with a N50 of 255.7 kb (Table 2). Nearly all genomic reads (>99%) were mapped back to the assembly while the map back rate of RNA-Seq reads was ~86%. BUSCO and CEGMA analysis suggested the whole genome sequence was reasonably complete with 91.6% complete and 3.6% partial BUSCO proteins present and 76.6% complete and 22.3% partial CEGMA genes present. The “scaffold13492” comprising 19,561 bp was identified as the *C. hookeri* mitochondrial genome. The total number of estimated archea, bacteria and virus scaffolds is only 1387 (Additional file 1).

**Table 2:** *Clitarchus hookeri* genome assembly statistics

	Size (bp)	Number of scaffolds
N90	188	859,481
N80	5446	48,684
N70	30,351	11,639
N60	148,642	5894
N50	255,691	3749
Longest	4,944,527	–
Total (>100 bp)	4,244,875,252	4,114,148
Total (>2 kb)	3,503,002,174	78,458

### Repetitive elements

The genome assembly of *C. hookeri* is highly repetitive, with approximately half the genome (51.6%) predicted as repeats. Among these, a total of 3210 repeat models were determined, 1404 (43.7%) of which were classified as different groups of interspersed transposon elements (TEs). To compare with *T. cristinae*, we used the same repeat identification method to identify repeats from the published genome assembly. In *T. cristinae*, 1288 repeat models were detected, including 433 (33.6%) assigned to the known repeat groups. The proportions of different repeat groups from the genomes of the two stick insects and the other two Polyneoptera species (*Zootermopsis nevadensis* and *L. migratoria*) [28, 59] are shown in Table 3.

**Table 3:** Comparisons of repeats among four Polyneoptera genomes

	*Zootermopsis nevadensis*		*Timema cristinae*		*Clitarchus hookeri*		*Locusta migratoria*	
Genome size (Mb)	490		1030		4240		6500	
Repeat types	Length (Mb)	P%	Lengh (Mb)	P%	Lengh (Mb)	P%	Length (Mb)	P%
DNA	12.78	2.60	75.13	7.30	612.35	14.43	1480.54	22.69
LINE	22.13	4.50	34.34	3.34	55.84	1.32	1332.72	20.42
LTR	0.72	0.15	5.89	0.57	103.59	2.44	508.68	7.80
SINE	9.78	2.00	42.88	4.17	84.08	1.98	141.18	2.16
Simple repeat	1.39	0.28	9.00	0.87	96.82	2.28	13.03	0.20
Other	0.00	0.00	1.00	0.10	0.74	0.02	0.03	0.00
Unknown	81.32	16.50	243.05	23.61	1237.41	29.15	406.10	6.22
Total	128.41	26.00	411.29	39.96	2190.84	51.60	3840.81	58.86

In the *C. hookeri* genome, miniature inverted repeat transposable element (MITE) was the most abundant repeat type, which was identified with 87 putative models and 1,214,018 copies, covering 5.79% of the assembly. The short sequence “rnd-1_family-8” was detected as the most frequent MITE repeat in the genome. Similarly, MITEs were also reported to be highly abundant in the stick insect *Bacillus rossius*, *B. grandii* and *B. atticus* partial genomes [20]. However, they were absent from the *T. cristinae* genome. In comparison, Maverick was the most abundant repeat type in the *T. cristinae* genome, predicted with 12 repeats comprising 145,675 copies, which cover 3.13% of the genome, and the most frequent repeat copy was “rnd-2_family-6”. Maverick was ranked as the third most abundant in the *C. hookeri* genome. The second most abundant DNA transposon in *C. hookeri* was TcMar-Tc1, the most abundant repeat type in the *L. migratoria* genome.

Class I TEs containing long terminal repeat retrotransposon (LTR), short interspersed element (SINE) and LINE were much less abundant than Class II TEs in the two stick insect genomes. Class I TEs constitute 5.74% and 8.08% of the *C. hookeri* and the *T. cristinae* genomes, respectively. LTR gypsy was the most abundant repeat type in the *C. hookeri* genome, comprising 194,787 copies, covering 1.44% of the genome. The short sequence “rnd-1_family-196” was detected with the highest copy number. In comparison, the LINE repeat RTE-BovB was the most abundant repeat type in the *T. cristinae* genome and the most frequent model was “rnd-3_family-716”. This repeat type was predicted to occupy 2.33% of the *T. cristinae* genome, comprising 75,215 copies. It is also one of the most frequent repeat types in the *L. migratoria* genome. There were only a few repeat types that were uniquely present in one of the stick insect genomes when compared with the other. The repeats RTE-RTE (LINE), ERV1 (LTR) and TRIM (LTR) were detected only from the *C. hookeri* genome, whereas Ngaro (LTR) was only present in the *T. cristinae* genome. The SINE repeats appear to be slightly more abundant in the *C. hookeri* than the *T. cristinae* genome.

### Protein-coding genes

**Fig. 1:**
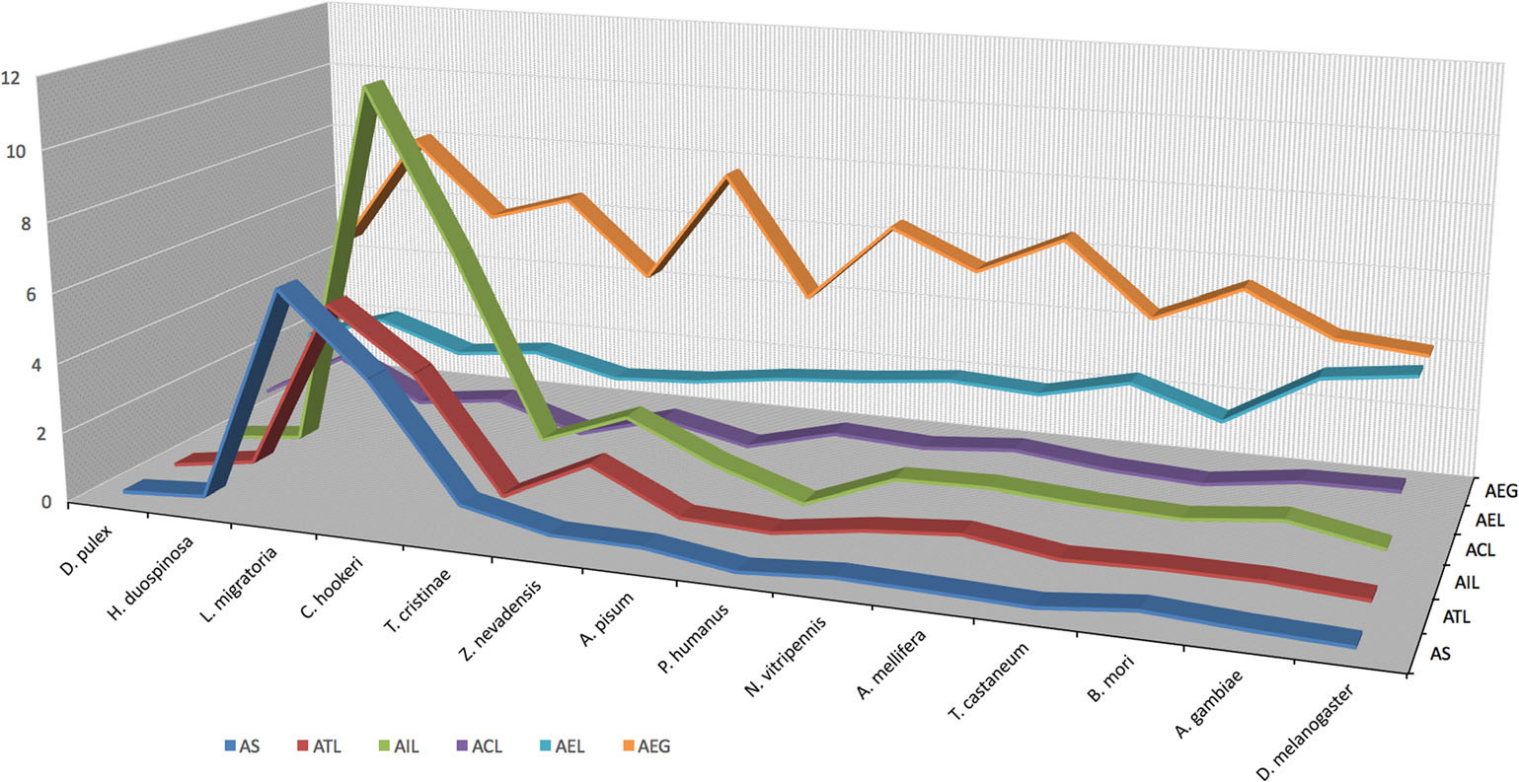
Correlation between genome size and gene model features across insects (*Holacanthella duospinosa*, *Locusta migratoria*, *Clitarchus hookeri*, *Timema cristinae*, *Zootermopsis nevadensis*, *Acyrthosiphon pisum*, *Pediculus humanus*, *Nasonia vitripennis*, *Tribolium costaneum*, *Bombyx mori*, *Anopheles gambiae* and *Drosophila melanogaster*) and *Daphnia pulex*. AS: genome assembly size; ATL: average of transcript length; AIL: average of intron length; ACL: average of coding region length; AEL: average of exon length; AEG: average number per gene. Scale numbering was adjusted by dividing ATL, AIL, ACL and AEL by 10^4^, 10^4^, 10^3^ and 10^2^, respectively

The current predicted 66,470 *C. hookeri* gene models include 10,266 models revised from transcript and protein sequences after in silico prediction. This gene set includes 779 (73.1%) complete predicted BUSCO proteins with 747 (95.9%) single-copy genes, and 154 (14.4%) partial proteins. The proportion of gene models that have Blast matches with *nr* database proteins was 36.2% (24,085), the majority of which hit the eusocial termite *Z. nevadensis* (42.3%), followed by the red flour beetle *Tribolium castaneum* (5.7%) (Additional file 2). There were 8478 predicted gene orthologues between the two sets of stick insect gene models using a reciprocal blast approach. We compared the average values of a variety of gene model features across *C. hookeri*, *T. cristinae*, *Holacanthella duospinosa* (New Zealand giant collembolan) [60] and a wide range of arthropods with these values available [28, 59] (Fig. 1). This analysis demonstrates that insects from Polyneoptera frequently have genome sizes in the gigabase range and that increases in genome size are positively correlated with increasing transcript and intron sizes.

### Gonadal transcriptome assembly and annotation

Approximately 95.8% of the 600 million raw RNA-Seq reads passed the cleaning criteria and were mapped to the genome assembly. The mapping ratios ranged from 89.8% to 94.9% across individuals and the ratios of uniquely mapped reads were all above 70% (Table 4). We then annotated only those gene loci with mapped RNA-seq reads, which can maximise the number of genes predicted to be expressed in the tissues compared with reusing the in silico produced gene models. In total, the mapped reads generated 39,940 putative gene loci, including 23,778 (59.5%) genes containing more than one exon, with strand orientation determined according to the intron-exon boundaries. Their transcribed sequences show an N50 length of 3828 bp, and a minimum and maximum length of 64 and 37,398 bp, respectively. A total of 36,072 (90.3%) transcripts have lengths longer than 500 bp. Only 45 sequences had the best homologous matches from bacteria and virus sequences within the Genbank *nt* database. This suggests an extremely low level (0.1%) of xenobiotic RNA contamination in this predicted gene set compared with our previous dataset generated from the same laboratory procedure [12].

**Table 4:** Genomic DNA mapping statistics

Sample ID	Read pairs	Uniquely mapped	No. of splices	Multiply mapped	Total mapped (%)
CLI765	47,699,036	37,219,558 (78.0%)	12,199,348	7,414,350 (15.5%)	93.6
CLI767	50,546,345	35,708,416 (70.6%)	11,153,462	12,243,359 (24.2%)	94.9
CLI768	45,978,461	34,815,789 (75.7%)	10,547,208	8,859,828 (19.3%)	95.0
CLI755	44,052,337	34,808,276 (79.0%)	10,253,623	6,884,797 (15.6%)	94.6
CLI757	45,000,013	32,997,927 (73.3%)	10,140,927	8,889,222 (19.8%)	93.1
CLI760	52,261,126	40,301,287 (77.1%)	13,724,663	6,631,109 (12.7%)	89.9

**Fig. 2:**
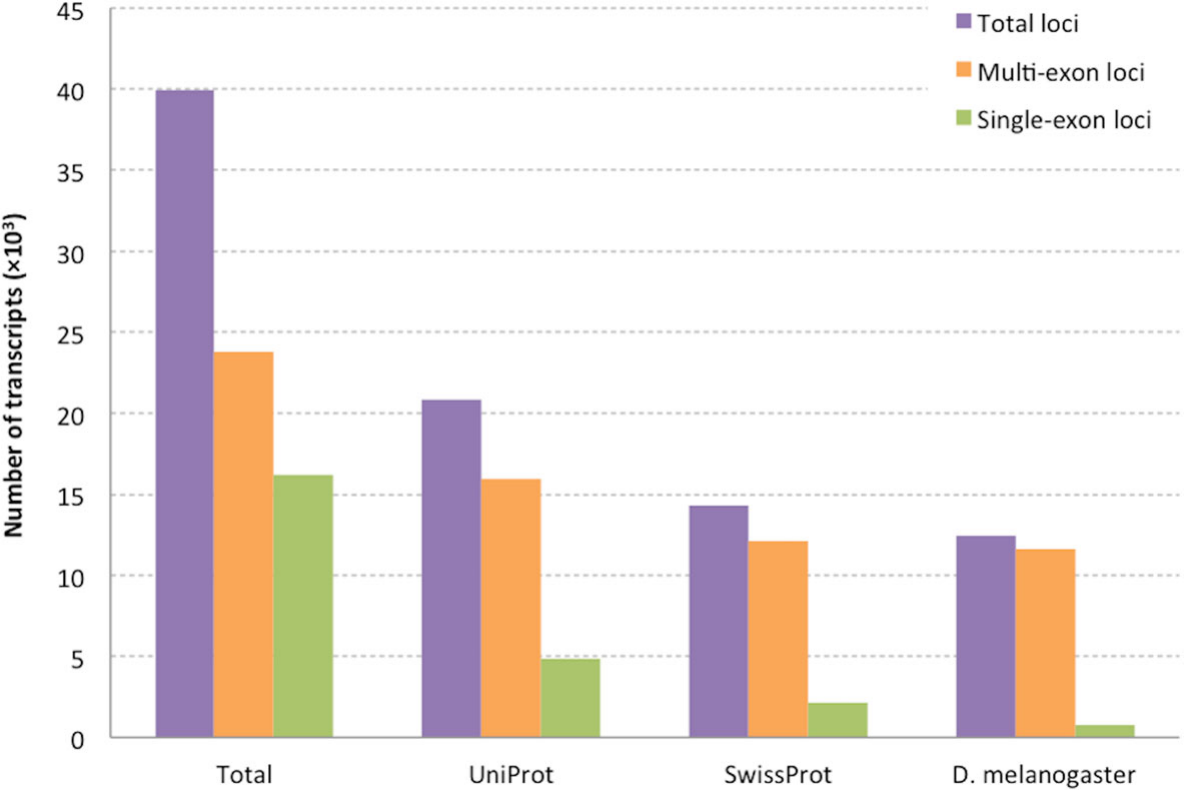
Distribution of homologous sequence matches from the Uniprot, SwissProt and *Drosophila melanogaster* protein databases, respectively

The number of transcripts having matches from the UniProt, SwissProt and *D. melanogaster* protein databases were 20,841 (52.24%), 14,270 (35.77%), and 12,443 (31.19%), respectively (Additional file 3). Among the UniProt blast hits, 37% were from the eusocial termite *Z. nevadensis*, followed by 7.5% and 4.5% from the pea aphid *Acyrthosiphon pisum* and the araneomorph spider *Stegodyphus mimosarum*, respectively. Notably, the proportion of single-exon transcripts having homologous matches was much lower than the multi-exon transcripts (Fig. 2), likely due to the fact that many of them were derived from non-coding regions, thus lacking similarity with sequences from protein databases.

### Gonadal sex-biased genes

**Fig. 3:**
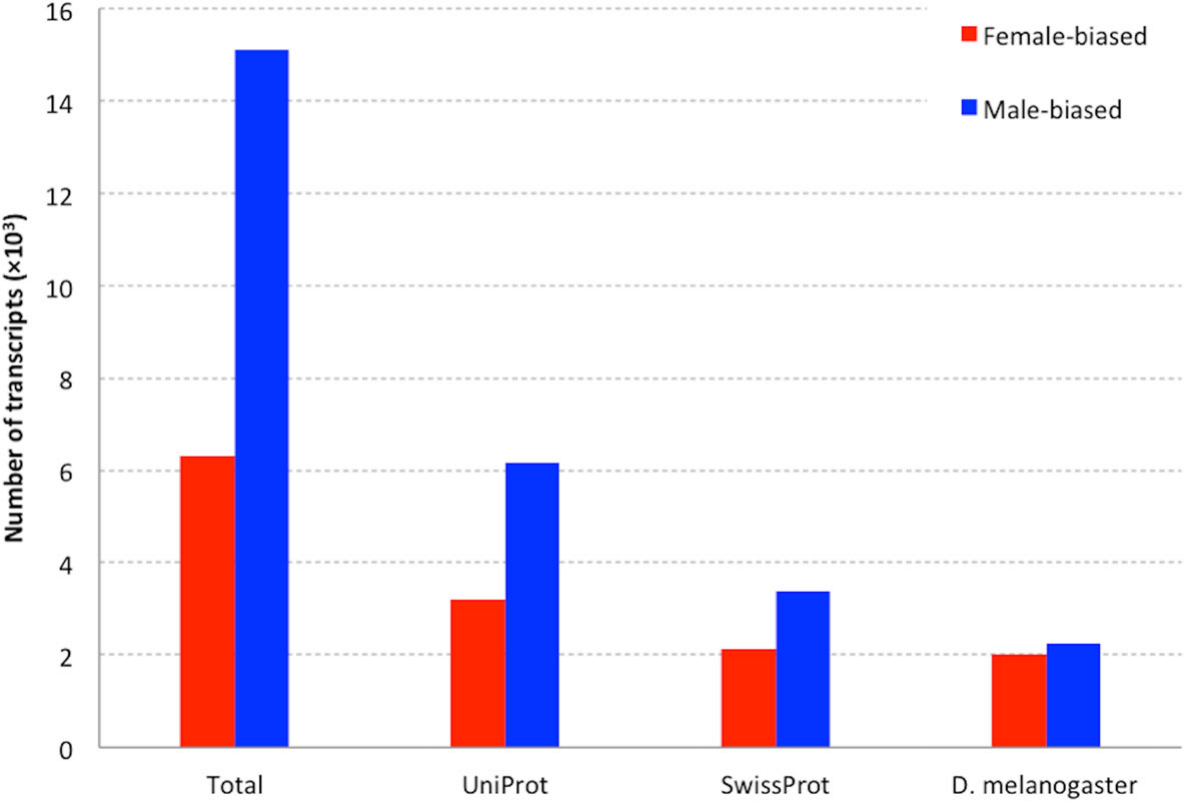
Distribution of homologous sequence matches from the Uniprot, SwissProt and *Drosophila melanogaster* protein databases for female-biased and male-biased genes, respectively

The female reproductive tract had 3262 gene loci that were uniquely expressed in this tissue, whereas approximately four times of this number (12,516) were detected in testis (ECS > 5). Comparative gene expression analysis between female and male gonadal samples revealed a large set of transcripts that were significantly differentially expressed (FC > 2 and BH adjusted *p* < 0.05). There were 6308 genes significantly overexpressed in the female, whereas the genes displaying male-biased expression had more than two-fold abundance (15,889) (Fig. 3 and Additional file 3). Notably, compared with female-biased transcripts, a much larger proportion of transcripts over-expressed in testis were lacking matches, especially when compared with the *D. melanogaster* protein database (Fig. 3).

The top 20 significantly over-expressed genes having *D. melanogaster* protein matches from each of the sexes are shown in Table 5. The genes with orthologues that were also highly expressed in *D. melanogaster* ovary were *CG1077*, *yellow-g*, *yellow-g2* and *Acph-1*, and testis were *CG12020*, *CG5458*, *CG32392*, *CG14838*, *CG13442*, *CG31068* and *CG17377*, respectively (data retrieved from the FlyAtlas Anatomy Microarray and modENCODE Anatomy RNA-Seq data on http://flybase.org).

**Table 5:** Summary of top 20 differentially expressed genes from female reproductive tract and testis, respectively

	Gene locus	Hit ID	Hit Name	E-value	Log2 FC	Adjusted p
Female-biased	XLOC_018823	FBgn0037405	CG1077^a^	2.38E-06	−13.06	1.58E-12
	XLOC_015696	FBgn0041709	yellow-g^a^	5.04E-19	−12.65	3.91E-11
	XLOC_032619	FBgn0266435	CG45065^a^	1.72E-151	−12.36	1.64E-10
	XLOC_030196	FBgn0035328	yellow-g2^a^	4.20E-65	−12.06	2.09E-36
	XLOC_007922	FBgn0035089	Phk-3	1.59E-08	−12.01	1.45E-91
	XLOC_009044	FBgn0034145	CG5065	1.75E-13	−11.93	2.21E-45
	XLOC_007550	FBgn0039084	CG10175	8.89E-79	−11.9	1.73E-50
	XLOC_021021	FBgn0038799	MFS9	1.96E-09	−11.89	1.59E-09
	XLOC_021022	FBgn0038799	MFS9	3.49E-45	−11.85	1.88E-21
	XLOC_025391	FBgn0013680	mt:ND2	6.59E-09	−11.81	1.74E-283
	XLOC_000499	FBgn0032433	Oatp33Ea	3.76E-32	−11.54	2.71E-09
	XLOC_003735	FBgn0034145	CG5065	3.59E-14	−11.52	6.18E-32
	XLOC_026733	FBgn0000032	Acph-1^a^	4.73E-69	−11.46	2.48E-41
	XLOC_002774	FBgn0000261	Cat	1.66E-34	−11.28	2.37E-08
	XLOC_005024	FBgn0039896	yellow-h	7.54E-111	−11.26	1.75E-18
	XLOC_024658	FBgn0030452	MFS10	5.42E-19	−11.23	2.92E-08
	XLOC_016572	FBgn0038799	MFS9	3.93E-29	−11.12	4.62E-08
	XLOC_032950	FBgn0000032	Acph-1^a^	3.86E-21	−10.99	6.21E-18
	XLOC_036184	FBgn0259247	laccase2	2.23E-09	−10.94	3.44E-09
	XLOC_018465	FBgn0026314	Ugt35b	6.37E-96	−10.91	4.59E-18
Male-biased	XLOC_037165	FBgn0014869	Pglym78	7.44E-70	13.61	1.81E-13
	XLOC_034828	FBgn0035273	CG12020^a^	9.64E-35	13.59	2.02E-13
	XLOC_033633	FBgn0020412	JIL-1	1.21E-19	13.47	4.23E-13
	XLOC_003842	FBgn0032478	CG5458^a^	3.75E-45	13.31	1.07E-12
	XLOC_039688	FBgn0038385	Fbxl7	6.21E-07	13.29	1.15E-12
	XLOC_035079	FBgn0019982	Gs1l	2.23E-53	13.29	1.19E-12
	XLOC_019177	FBgn0020412	JIL-1	2.99E-16	13.25	1.45E-12
	XLOC_039094	FBgn0052392	CG32392^a^	2.03E-08	13.12	3.14E-12
	XLOC_010029	FBgn0005612	Sox14	3.45E-08	13.05	4.59E-12
	XLOC_016670	FBgn0033635	CG7777	2.72E-26	12.98	2.59E-27
	XLOC_035177	FBgn0004380	Klp64D	1.96E-09	12.94	1.30E-35
	XLOC_024216	FBgn0264574	Glut1	1.89E-43	12.88	8.30E-25
	XLOC_004823	FBgn0035799	CG14838^a^	1.28E-72	12.84	1.01E-25
	XLOC_010324	FBgn0034546	CG13442^a^	2.57E-10	12.84	1.45E-11
	XLOC_019341	FBgn0036211	CG5946	9.38E-16	12.77	2.02E-11
	XLOC_002845	FBgn0020412	JIL-1	6.64E-13	12.75	2.79E-24
	XLOC_035152	FBgn0051068	CG31068^a^	1.24E-33	12.73	2.62E-26
	XLOC_038804	FBgn0031988	CG8668	1.07E-63	12.65	7.43E-25
	XLOC_020898	FBgn0031859	CG17377^a^	2.27E-10	12.58	1.47E-24
	XLOC_038151	FBgn0039396	CCAP-R	6.78E-28	12.57	5.83E-11

“^a^” indicates the transcript is also highly expressed in the *Drosophila melanogaster* ovary and testicle respectively

### Enriched gene ontology terms and pathways

**Fig. 4:**
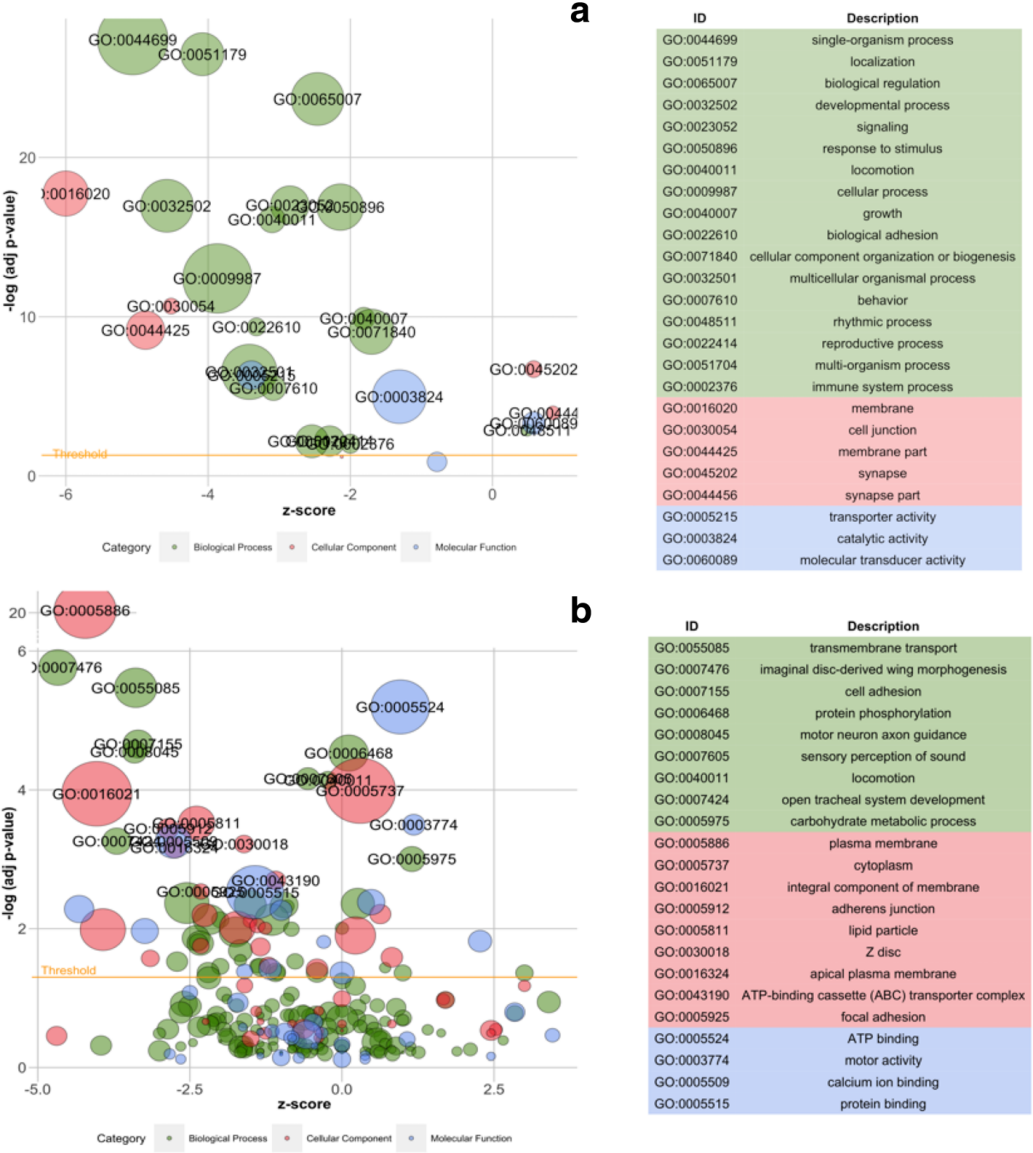
Bubble plots showing enriched GO terms generated from sex-biased genes. “Z-score” was calculated for each term using the formula: male-biased minus female-biased gene number divided by the square root of sex-biased gene number. The significant GOs are indicated above the yellow line. The bubbles calculated with minus z-scores represent GOs containing more female-biased genes, while GOs with more male-biased genes have z-scores larger than zero. The bubble size represents the number of genes. The GO descriptions on the right are listed from the highest to lowest significance of enrichment. (**a**) Level one terms; (**b**) after removing all parental terms

The matched *D. melanogaster* protein hits from the *C. hookeri* sex-biased genes were used to detect enriched gene ontology (GO) terms. In summary, a total of 2405 *D. melanogaster* FlyBase IDs were subjected to multiple tests of GO enrichment using DAVID. Within level one GOs, most of the significantly enriched terms were female-biased. Some of these terms closely represent female features of reproduction, such as developmental process, signaling, reproductive process and immune system process from the biological process category (BP). In comparison, rhythmic process (BP), molecular transducer activity (MF) and synapse (CC) were significantly enriched among male-biased genes (Fig. 4a).

After removing all the parental terms, the more specific GOs were revealed (Fig. 4b). The significantly enriched terms for the male-biased genes include ATP binding, motor activity, microtubule binding and ATPase activity (MF), corresponding to sperm maturation and movement; whereas, the terms for the female-biased genes include calcium ion, protein and actin binding and ATPase activity (MF). In addition, females also over-expressed genes involved in glucose transmembrane transporter activity, oxidoreductase activity and carboxylic ester hydrolase activity (MF) (Fig. 4b). Furthermore, we also found GOs associated with oocyte maturation and development, such as imaginal disc-derived wing morphogenesis, open tracheal system development (BP), myofibril assembly, neuron projection morphogenesis and dorsal closure (CC) that were enriched within female-biased genes.

**Fig. 5:**
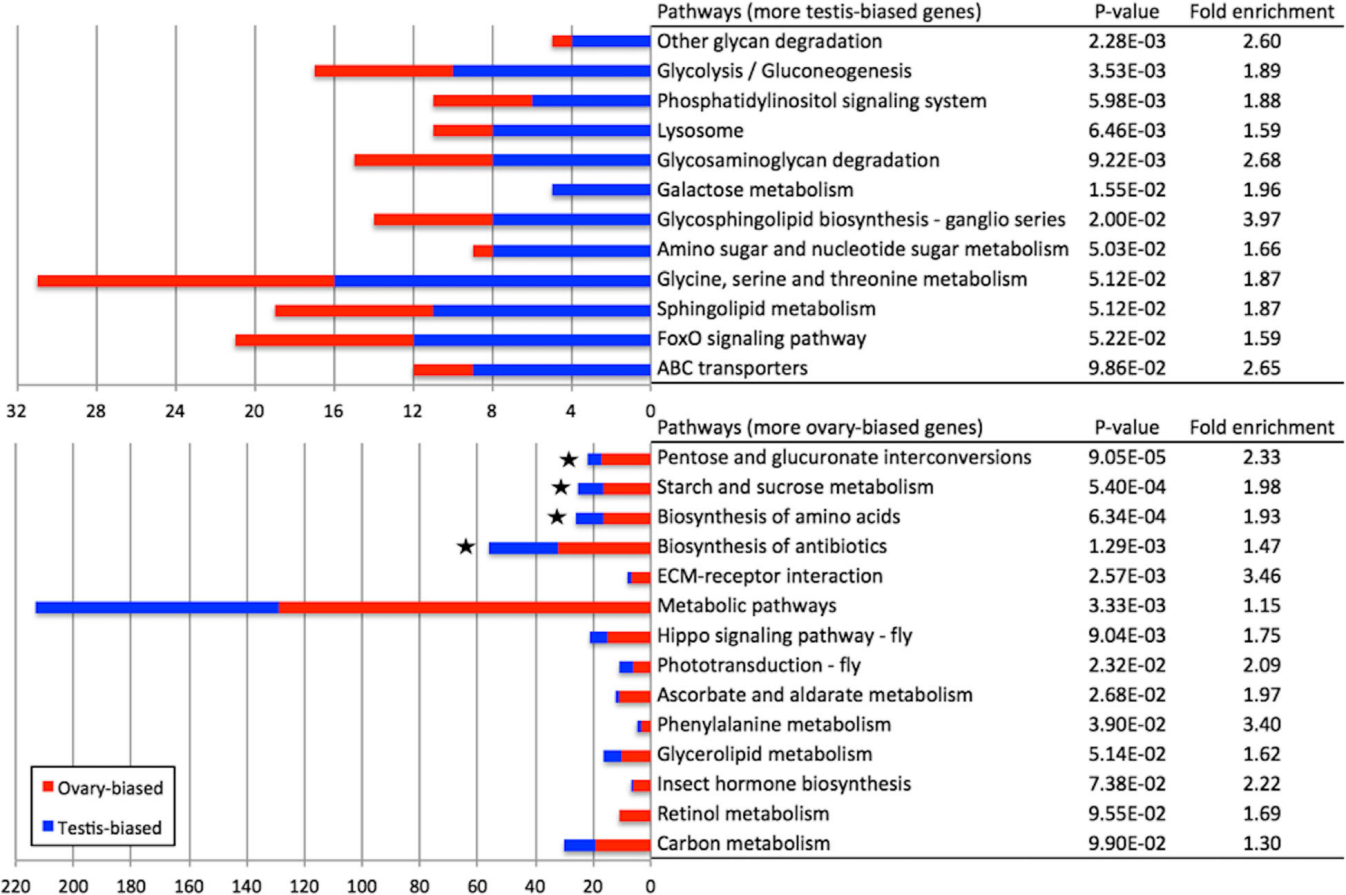
KEGG pathways enriched in gonadal sex-biased genes. Enriched pathways with modified Fisher exact test *p* value below 0.1 are shown here. Stars indicate pathways with BH adjusted *p* value below 0.05

The enriched KEGG pathways include many related to carbohydrate metabolism, including pentose and glucuronate interconversions and starch and sucrose metabolism enriched within female-biased genes, and glycolysis/gluconeogenesis, glycan degradation, glycosaminoglycan degradation and galactose metabolism pathways enriched within male-biased genes (Fig. 5). Other enriched pathways within female-biased genes were ECM-receptor interaction, hippo signaling pathway, phototransduction, insect hormone biosynthesis and retinol metabolism, and within male-biased genes were lysosome, phosphatidylinositol signaling system, glycosphingolipid biosynthesis, foxO signaling pathway and ABC transporters (Fig. 5). Within the juvenile hormone (JH) biosynthesis pathway (belonging to the insect hormone biosynthesis pathway), JH epoxide hydrolase 2 (*Jheh2*) and *CG9360* were over-expressed in females.

## Discussion

The *C. hookeri* genome is the second largest insect genome published to date after *L. migratoria* [28]. It is also the second stick insect genome to be reported and the only one sequenced from the Euphasmatodea. Thus, the availability of this genome sequence provides valuable information to investigate the evolution of large insect genomes and the diversification of the Phasmatodea. In particular, parthenogenesis and other alternative reproductive strategies have played an important role in phasmid evolution. However, the molecular mechanisms underlying parthenogenesis have been poorly studied. Characterisation of genes essential to stick insect reproduction, especially from species with different reproductive modes, is the first step towards understanding reproductive flexibility. In this study, we constructed a *C. hookeri* genome assembly, which was then used as a reference genome to detect genes that are present in the female reproductive tract and testicle transcriptomes.

### Evolution of repeats and gene length expansion

The *C. hookeri* genome assembly comprising 4.2 Gb, was very similar to the flow cytometry estimate, and approximately four times larger than *T. cristinae* [19]. Similar to *L. migratoria*, the analysis of the *C. hookeri* genome also revealed a large volume of repetitive elements. DNA transposon (Class II) was the most dominant repeat group in the stick insect and the *L. migratoria* genomes; whereas, the genome of *Z. nevadensis*, only around one third of *T. cristinae* genome size, was mostly enriched with Non-LTR retroid long interspersed element (LINE). The comparison also shows that the increasing genome size from *Z. nevadensis* to *L. migratoria* is consistent with the growing absolute amount and proportion of DNA transposons. Relative to *T. cristinae*, the *C. hookeri* genome is expanded with a variety of repeat types. Notably, MITE, the most abundant group of DNA transposon in the *C. hookeri* genome assembly, was also reported to be present at [61] high frequency in the partial *Bacillus* genome sequences [20], but absent in the *T. cristinae* genome. Either the invasion and proliferation of MITEs occurred along the evolution of euphasmids or the loss of MITEs occurred along the *Timema* lineage. A survey of MITEs across a wide range of stick insect lineages may shed light on the evolution of this repeat group in stick insects. A relatively large proportion of un-classified repeats were also revealed in the two stick insect genomes, whether they indeed represent novel repeat families requires further analysis.

The annotated gene models from *L. migratoria* and *C. hookeri* were roughly 6 and 5 times longer than other insects with moderate to small genome sizes. This suggests there was an intron length expansion during the formation of large insect genomes. There are several consequences to an increase in intron length. First, long introns are associated with an increased metabolic cost as introns are transcribed together with exons [61]. Second, they are thought to be associated with the large genome chromosomal compactness [61]. Third, they may be negatively correlated with recombination [62]. The *C. hookeri* genome provides a novel resource to test these three hypotheses in insects. In addition to longer introns, the *C. hookeri* genes also exhibited a higher number of introns and exons per gene and a slightly longer coding sequence length compared with *T. cristinae*. However, whether these patterns hold across the Euphasmatodea requires the availability of more stick insect genome sequences.

### Sex-biased genes

The comparative transcriptome analysis between female and male gonads revealed a large set of genes with sex-biased expression. The male-biased genes were more than twice as abundant as those displaying female-biased expression, and a much greater proportion of male-biased genes were lacking blast matches from the known proteins. Also, *Clitarchus hookeri* females sometimes reproduce by parthenogenesis, which might lead to less selective pressure on males because the male-biased genes are used less frequently than the female-biased genes [63, 64]. It has also been reported that the sexually-derived *Timema* parthenogenetic lineages have experienced sexual trait decline, such as shrunken spermatotheca and the loss of male attractiveness [65]. This has also likely occurred in the *C. hookeri* parthenogens. It is possible that this sexual trait decay correlates with female-biased gene expression change. All these hypotheses require further investigation of the sequence and expression divergence across euphasmids and the gene expression between sexual and parthenogenetic *C. hookeri*.

The *C. hookeri* genes that are over-expressed in female reproductive tract are enriched in development, signalling, growth, behaviour and reproductive process. The top five female-biased DE genes had *D. melanogaster* matches with a chorion-containing eggshell formation protein (*CG1077*) predicted to have anti-microbial activity [66], *yellow-g* and *yellow-g2* proteins essential to eggshell integrity [67], *CG45065* protein responding to mating [68], and *Phk-3* protein associated with metamorphosis [69]. Stick insects show interesting traits relating to egg morphology [70, 71]. Eggs have in a variety of shapes, often mimicking plant seeds, many of which contain a knob-like capitulum that resembles an elaiosome to attract ants for burial [72]. Recently, a species (*Korinninae* sp.) was found to produce an ootheca containing numerous eggs in a highly ordered arrangement, which is distinct from other stick insects that produce eggs singly by dropping them to the ground or inserting them into crevices or soil [73]. The genes identified in our study may be essential to eggshell formation and maturation and are candidates to further investigate egg variation and adaptations in stick insects.

In comparison, the male-biased genes were significantly enriched for rhythmic process, molecular transducer activity and synapse. Rhythmic processes play important roles in temporally coordinating the release of sequential sperm, the acidification of the vas deferens, and contractile activity [74–77]. The enrichment of the molecular transducer activity was also found in crab and a sex-changing fish testis [78, 79]. The top five male-biased genes with blast matches include a glycolysis protein *Pglym7*8 that is present in the semen and seminal vesicle tissue of a honey bee [80], *CG12020* enriched in the sperm proteome with a role in protein folding [81, 82], *JIL-1* essential for chromosomal organisation [83], *CG5458* involved in sperm axoneme assembly [81, 82] and *Fbxl7* regulating mitosis through Aurora A [84].

Notably, a large number of sex-biased transcripts showed no matches with any of the databases. These transcripts may include: 1) highly diverged genes; 2) unknown genes; and 3) non-coding elements. Stick insects contain panoistic ovaries where oogonia eventually differentiate into oocytes [85]. However, insect ovary-biased genes have mostly been identified from the meroistic ovaries frequently present in the derived Holometabola, where oogonia differentiate into an oocyte and several nurse cells [86, 87]. Thus, some of the novel female-biased genes found in this study may be playing unique functions in the panoistic ovary.

Interestingly, we found some *C. hookeri* genes with matches to *D. melanogaster* proteins involved in the JH synthesis pathway. These genes include *Jheh1* and *Jheh2* involved in the JH catabolic process [88] and *CG9360* having an oxidoreductase activity [89], *Jheh2* and *CG9360* over-expressed in female *C. hookeri*. It has been suggested that in the cyclical parthenogenetic aphids, higher levels of JH induce parthenogenetic reproduction [90–93]. However, whether the level of JH has an impact in the differentiation of the southern parthenogenetic *C. hookeri* is unknown. This could be examined by assessing levels of JH or indirectly by measuring expression levels of the genes involved in JH hormone biosynthesis. In addition, we also found some enriched pathways were related in carbohydrate metabolism (e.g. starch and sucrose metabolism and glycan degradation), which are likely to contain genes playing important roles in nutrition and energy support for the production of gametes and the maintenance of reproductive organs.

### Conclusions

The analysis of the *C. hookeri* genome assembly revealed a large, repetitive genome, likely resulting from the accumulation of DNA transposons along with an increase of intron length. MITE, the most abundant repeat type in the *C. hookeri* genome assembly, contributed significantly to the growth in genome size. Using a reference-guided approach coupled with differential expression analysis, a large number of sex-biased genes were identified by comparing gonadal transcriptomes between females and males. Female reproductive tract over-expressed genes were involved in development, signalling, growth, behaviour and reproductive process, whereas, testicle over-expressed genes were involved in rhythmic process, transducer activity and synapse. We also identified several genes involved in JH synthesis that were over-expressed in the female. These genes are an important resource for furthering understanding of the evolution of reproductive strategies within Phasmatodea.

## Additional files

Additional file 1: Scaffolds match archea, bacteria and virus sequences. (XLSX 140 kb)

Additional file 2: BLAST results from gene models. (XLSX 5388 kb)

Additional file 3: BLAST results from transcripts, raw counts from gene expression and differential expression results. (XLSX 13185 kb)

## Abbreviations

Bp: Base pair
BP: Biological process
CC: Cellular component
Gb: Gigabases
GO: Gene ontology
JH: Juvenile hormone
Jheh2: JH epoxide hydrolase
LTR: Long terminal repeat retrotransposon
MF: Molecular function
MITEs: Miniature inverted repeat transposable elements
MP: Mate pair
PE: Paired end
SINE: Short interspersed element
TEs: Interspersed transposon elements
